# Learning a Practical Psychotherapeutic Skill in Higher Education in Sweden: A Conceptual Paper Concerning the Importance of Constructive Alignment When Teaching Therapeutic Alliance

**DOI:** 10.32872/cpe.12037

**Published:** 2024-09-30

**Authors:** Ann-Sophie Lindqvist Bagge, Rolf Holmqvist, Therése Skoog, Malin Hildebrand Karlén

**Affiliations:** 1 Department of Psychology, University of Gothenburg, Gothenburg, Sweden; 2 Department of Behavioral Sciences and Learning, Linköping University, Linköping, Sweden; University of Vienna, Vienna, Austria

**Keywords:** constructive alignment, clinical psychology training, psychology education, therapeutic alliance

## Abstract

**Background:**

In addition to theoretical education, clinical psychology programs should include practical skills training. This skill training may be tied to specific assessment and treatment methods; other skills, such as the ability to create a collaborative alliance with patients, are more generic. Previous research has shown that the ability to build a therapeutic alliance (TA) is often not systematically taught in clinical psychology programs and it is uncertain how this competence is examined. A lack of competence in establishing TA on the part of the psychologist might diminish the effects of psychotherapy. To meet the Bologna Declaration, European universities need to demonstrate constructive alignment, i.e. a relationship between elements of the course content and intended learning outcomes in course documents, and show how the acquired knowledge, abilities, and approaches are assessed.

**Method:**

This conceptual paper reviewed the syllabuses for universities in Sweden offering the five-year clinical psychology program to illustrate how higher education in Sweden adheres to the Bologna recommendation on constructive alignment when teaching TA to future clinical psychologists.

**Results:**

Only two universities out of all eleven universities in Sweden offering a psychology program described satisfactory constructive alignment concerning TA.

**Conclusion:**

This conceptual paper raises awareness of the importance of pedagogic structure when teaching TA in higher education by pointing to the prevailing lack of constructive alignment in teaching TA. The increased awareness will hopefully lead to improved structuring in the teaching of TA.

## Highlights

- Shortcomings in TA training may reflect broader challenges in psychotherapy education across European universities.
- TA training should be a core element of future clinical psychology program curricula.
- European institutions are encouraged to integrate TA training consistently into curricula and ILOs.

## Background

Research and theoretical reflection about the significance of general relational factors in contrast to more specific techniques and methods in psychological treatment have been in focus for decades (Norcross & Wampold, 2019). Recent theoretical and empirical work indicates a complex interaction between specific and general relational factors for patient outcomes (Heinonen & Nissen-Lie, 2020; Lorenzo-Luaces & DeRubeis, 2018; Uhl et al., 2022; Webb et al., 2010). Considering the importance of relational factors, it is interesting that professional training and clinical experience do not seem to improve the competencies needed to increase patient outcomes (Christensen & Jacobson, 1994). Hayes and colleagues (2022) recently commented on “the paradox that experience in psychological intervention reliably leads to increases in confidence but not in competence” (p. 19). In parallel with this discussion, the issue of the significance of therapists’ adhering to treatment integrity has also evoked research interest. Although the findings on this issue are heterogeneous, it seems apparent that general relational factors contribute substantially to patient change (Heinonen & Nissen-Lie, 2020; Uhl et al., 2022; Webb et al., 2010) The question of how to combine technique with relational competence is complex (Seewald & Rief, 2023).

Studies on Facilitative Interpersonal Skills (FIS; Anderson et al., 2016) have shown that factors like a therapist’s capacity for establishing warmth, persuasiveness, hopefulness, and ability to create an alliance and repair problems are associated with more positive treatment outcomes. Bennett-Levy summarized recent studies about the role of the therapist by stating that effective therapists are characterized by “the relational qualities and skills associated with alliance building and maintenance, and cognitive and emotional personal qualities such as resilience, mindfulness, tolerance of ambiguity, self-confidence, healthy self-doubt, capacity for self-reflection and self-awareness” (Bennett-Levy, 2019, p. 141).

### Therapeutic Alliance (TA)

The most studied relational factor is the therapeutic alliance (TA) (Bordin, 1979; Wampold, 2015). The usual definition implies that TA consists of three components: (1) goal – the therapeutic purpose, (2) task – the therapeutic process, and (3) bond – the therapeutic relationship (Bordin, 1979). The creation of the alliance can be seen as a joint effort by the patient and therapist. The alliance contributes significantly to the effects of psychotherapy (Flückiger et al., 2018). The explicit aspect of TA concerns agreement and negotiations about goals and tasks, whereas the bond aspect of TA comprises the patient's emotional ties to the therapist and the therapist's empathetic ability and involvement in the patient's situation (Summers & Barber, 2003). A meta-analysis (Del Re et al., 2021) concludes that average differences between therapists in the alliance with their patients have a stronger influence on outcomes than differences between patients within therapists. This finding underscores the importance of stimulating therapists to improve their ability to create constructive cooperation with the patient.

Studies have shown that the quality of the TA contributes to treatment outcomes (Flückiger et al., 2018; Summers & Barber, 2003). A two-stage individual meta-analysis showed reciprocal within-patient correlations between higher ratings of alliance and lower symptom load in the first seven sessions of psychotherapeutic contact, indicating that better TA contributes to symptom reduction (Flückiger et al., 2020). The authors concluded that at least in the early phase of psychotherapeutic contact, symptoms, and alliance were reciprocally related to one other, generating a positive spiral, increasing the sense of alliance, and lowering symptom load in the subsequent sessions (Flückiger et al., 2020). The causal relationship between alliance and session outcome is complex. Most studies do, however, find that the primary causal link is from better alliance to better outcomes (Crits-Christoph & Gibbons, 2021). The alliance-outcome relationship is a key factor across different psychotherapeutic treatment approaches (Flückiger et al., 2018). This is also valid for patients with personality disorders and severe mental conditions (Caspar, 2019).

### Can TA Be Taught?

Summers and Barber (2003) concluded that: (1) the ability to develop TA can improve during training, (2) trainees become more focused on TA with accumulated training and complex case formulations, (3) the goal and task aspects of TA may be more learnable and teachable than the bond aspect, and (4) there are preexisting therapeutic factors that affect the ability to develop TA (Summers & Barber, 2003). Summers and Barber (2003) further argue that of the three factors likely to influence the development of TA, namely patient characteristics, therapist characteristics, and the therapist's technical activity during treatment, it is probably the therapist's technical activity that is most susceptible to training. Zilcha-Mano (2021) points to the importance of distinguishing between trait-like and state-like aspects of the therapist’s contribution. Therapists need to be able to distinguish between their trait characteristics and their reactions which are more situational (Zilcha-Mano et al., 2019).

Ever since Delaney and Heiman (1966) and later Grace et al. (1995) showed that trainees could be taught increased sensitivity to non-verbal communication, several studies have shown positive outcomes of various forms of alliance training. Crits-Christoph and colleagues (2006) found in a small study with five therapists that TA training improved the quality of therapist- and patient-rated TA, but not patient outcomes. Another study found that guidance from a supervisor that focused on TA ability or the alliance process was associated with a reduction in psychiatric symptoms and retention of positive outcomes in patients in therapy – these patients evaluated the therapy more positively at the end of treatment (Bambling et al., 2006). In still another alliance training study, Smith-Hansen et al. (2011) found improved alliance but no patient outcome effects. Recently, interest has been focused on the restoration of alliance ruptures. Studies have found that therapies where alliance ruptures are repaired attain better outcomes (Eubanks-Carter et al., 2015; Larsson et al., 2018; Safran et al., 2002). For example, The Alliance-Focused Training program (AFT) (Eubanks-Carter et al., 2015; Safran & Muran, 2000; Safran et al., 2011) is based on studies of alliance ruptures and their reparation. This program focuses on training self-awareness, affect regulation, and interpersonal sensitivity to increase therapists’ awareness of strains in the alliance and competence in repairing conflicts and ruptures. Recently, the Facilitative Interpersonal Skills (FIS) model has been combined with the AFT to create another training model, the AFT/FIS training (Perlman et al., 2023). The Personal Practice model created by Bennett-Levy also contains elements that focus on alliance-building (Bennett-Levy & Finlay-Jones, 2018). Studies of the results of skills training of relational variables show varying results. In a research overview, Knox and Hill (2021) conclude that although some persons may have more talent than others for psychotherapy, skills training does improve performance.

Regardless of the specific programs used in training to increase prospective therapists' TA competence, Summers and Barber (2003) recommended the following pedagogical approaches to improve TA: (1) early didactic and tutoring focus on TA concept and its techniques, (2) sustained attention on TA throughout the students' practical clinical TLAs (case formulations, conceptualizations, providing clinical tutor guidance in expected alliance ruptures within the discussed cases, etc.), (3) didactic and tutorial focus on establishing appropriate, realistic, and discussed goals and on identifying the patient’s and therapist's tasks in the therapy context, and (4) the concept ‘to develop TA’ should be integrated with clinical data regarding TA in case formulations and conceptualizations when teaching TA (Summers & Barber, 2003). In addition to the authors' belief that TA should be a central learning aspect of clinical psychology programs given its significant role as TA in psychological treatment, it is important to include clinical practitioners as lecturers when teaching practical skills in higher education courses to obtain a constructive balance between theory and practice (Williams & Joyce, 2009).

## The Pedagogic Necessity of Constructive Alignment (CA)

As the therapist’s ability to establish and consolidate TA is a significant factor in treatment outcome, it is important to ascertain if and how this skill is taught in clinical psychology courses. It could be expected that training in TA would be a central learning target in clinical psychology programs. As early as 1990, it was suggested that training therapists should attend to the interpersonal processes in treatment relationships (Alberts & Edelstein, 1990). Studies have found, however, that TA is often not taught systematically in higher education (Constantino et al., 2017; Constantino et al., 2013). Since TA is usually not taught systematically there is an obvious need for a pedagogic focus on how TA is taught. If this pedagogic need is not met, graduate clinical psychologists could leave higher education without sufficient TA skills, despite research showing the importance of improving TA during the education of prospective psychologists (Grace et al., 1995).

### Constructive Alignment (CA)

To meet the European aim of establishing general standards of teaching in higher education, i.e., The Bologna Declaration of 19 June 1999. Joint Declaration of the European Ministers of Education (European Higher Education Area [EHEA] and Bologna Process, 1999), universities need to demonstrate a logical relationship between elements of the course content and intended learning outcomes (ILOs) in course documents and how the acquired knowledge, abilities, and approaches are assessed (EHEA, 2015; González & Wagenaar, 2003).

The pedagogic idea that a constructive link, or alignment, should exist between ILOs, Teaching and Learning Activities (TLAs) and Assessments was developed by Biggs (1999). According to Biggs, constructive alignment makes explicit the standards needed if the ILOs are to be achieved and maintained. The underlying principle of constructive alignment is that the assessment tasks should comprise an authentic representation of the ILOs (Biggs & Tang, 2011). Constructive alignment could be used as a theoretical tool for planning TLAs and assessment tasks that aim to directly address the ILOs (Biggs & Tang, 2011), where the TLAs include what the teacher does (teaching activities) and what the student does (learning activities). ILOs did not feature in the original Bologna Declaration of 1999 but were included in the 2003 Berlin Communiqué and have since become the core component for evidencing qualifications at the European level, CA has been explicitly referenced from 2015 onwards (Hailikari et al., 2022; Loughlin et al., 2021). CA is today the foundation for the current standards and policies for program specification, and declarations of ILOs, in Europe (Fransson & Friberg, 2015; Ruge et al., 2019; Schmidt, 2019) since the establishment of the European Higher Education Area in 2010 (EHEA, 2021) and the Standards and Guidelines for Quality Assurance in the European Higher Education Area (ESG) in 2015 (ENQA et al., 2015). The ECTS (The European Credit Transfer and Accumulation System) Users’ Guide is a tool of EHEA that specifies the responsibility of university teachers to ensure that the constructive alignment of ILOs, TLAs, and assessment is “an essential requirement for educational programmes” (European Commission, Directorate-General for Education, Youth, Sport and Culture, 2015).

### An Illustrative Example From Swedish Higher Education

Despite the recommendations of the Bologna process, there is much heterogeneity between European countries regarding clinical psychology training (Laireiter & Weise, 2019). Sweden has been a full member of the Bologna Process/European Higher Education Area since 1999. The Bologna declaration and constructive alignment are highly relevant to the Swedish clinical psychology program. To practice as a clinical psychologist in Sweden, the student must complete five years of master’s level university studies in the national Psychology Program (Master of Science in Psychology, 300 European Credit Transfer and Accumulation System [ECTS]) and then complete one year of practical service under continuous supervision (*Swe.* Praktisk Tjänstgöring för Psykologer [PTP]). After approved PTP, the student is granted a license as a clinical psychologist by the Swedish National Board of Health and Welfare (https://www.government.se/government-agencies/national-board-of-health-and-welfare--socialstyrelsen/) and the psychologist can then practice clinical psychological treatment without supervision in private settings or within the Swedish public healthcare system. During the 5-year psychology program, the clinical psychotherapy courses are often taught during the latter part of the program. The psychotherapy courses are taught separately or as integrated into other courses in the psychology programs. During the psychotherapy courses, the psychology student is taught theoretical psychotherapeutic knowledge and is allowed to practice this knowledge during supervised psychotherapeutic treatment with patients with milder forms of psychological problems. To become a licensed psychotherapist in Sweden, as opposed to a licensed clinical psychologist, students who have taken the clinical psychology program must also complete another 3-year program (a graduate diploma course in psychotherapy).

This conceptual paper aimed to illustrate how higher education in Sweden adheres to the Bologna recommendation on constructive alignment when teaching TA to future clinical psychologists. To assess the extent of training in TA, we observed how TA was taught regarding CA. A country-wide overview of the course syllabuses and ILOs of TA-relevant psychotherapy courses in the clinical psychology program was performed in 2019, by two of the authors, for all eleven universities in Sweden offering the five-year clinical psychology program. The two authors analyzed independently of each other the presence of explicit mention of the term TA (or its synonyms cf. working alliance, treatment alliance, etc.) based on the definition of Bordin (1979) in the TA-relevant clinical psychotherapy courses’ syllabuses and ILOs.

The reviewed document analysis showed that out of the eleven universities in Sweden offering a clinical psychology program, only two universities specifically stated, ‘therapeutic alliance’ (or its synonyms) in the ILOs in their course syllabuses. At nine universities, different aspects of the concept of TA were in some (vague) way described in the ILOs. When TA (or its synonyms) is not mentioned in the courses’ ILOs and when imprecise TA definitions do not fully correspond to the established definition of TA, a constructive link between ILOs, TLAs, and assessment/examination when teaching TA cannot be said to exist. The obvious lack of CA was due to an absence of a clear and explicit description of TA (or its synonyms) in the ILOs of the clinical courses for nine out of the eleven universities in Sweden offering a clinical psychology program. Definitions of TA should be operationalized in the ILO for programs to be able to teach and examine the students’ TA abilities/knowledge when applying the pedagogic concept of CA. The present illustrative example shows that teaching – and training – of TA is not done systematically in Swedish universities when training clinical psychology students’ ability to create a viable and constructive cooperative alliance with patients. As the data collection was conducted in Sweden only, the results cannot be generalized to universities in other European countries. The results can, however, hopefully serve as an illustration of a pedagogical problem that has been described in scientific journals (Constantino et al., 2017; Constantino et al., 2013) as well as has been informally discussed among colleagues internationally.

## Conclusion

Teaching TA is difficult as it concerns a relational and, to a large extent, implicit skill. Nevertheless, this skill – alliance building and alliance maintenance – is a precondition for effectively using specific methods in psychotherapy. At the same time, psychotherapy is a craft as well as a science, and training programs need to ascertain that the student has acquired sufficient TA competence both theoretically and practically. The craft aspect of TA may be challenging to conceptualize in traditional university teaching contexts. We fear that the illustrative example of the current paper – showing vague descriptions of TA and a lack of constructive alignment between course objectives, ILOs, and examination methods – reflects a situation where TA is not systematically taught in clinical psychology programs in Sweden. Considering the significance for psychologists and their future patients of developing competence in alliance-building, preferably through constructive alignment, and the findings in this study, it is doubtful whether the current structure for teaching TA at Swedish universities offers prospective clinical psychologists an opportunity to develop sufficient TA knowledge and skills. A lack of basic TA competence in this might diminish the efficacy of psychotherapy given by future clinical psychologists. This conceptual paper hopefully directs the focus on the structure around the teaching of practical psychotherapeutic skills in higher education, illustrated by the lack of constructive alignment when teaching TA to clinical psychology students in higher education. It is also hoped that this conceptual paper will stimulate improved structuring of the teaching of TA. Based on the backdrop of the current conceptual paper – and given the important role of TA in enhancing the effectiveness of treatment and fostering positive outcomes for patients – our strong recommendation is to make alliance-building training a core element of future clinical psychology program curricula in Sweden. A good way to start is with TA being specifically mentioned in the ILOs. The importance of TA in clinical psychology education resonates with the ESG's emphasis on fostering student-centered learning outcomes and ensuring education effectiveness (ENQA et al., 2015). The identified shortcomings in TA training may reflect broader challenges in psychotherapy education across European universities, not just in Sweden. We encourage European institutions to adhere to the ESG principles by integrating TA training consistently into curricula and aligning it with program ILOs. Such a strategic approach ensures that future clinical psychologists across Europe receive comprehensive training in TA, promoting quality in psychotherapy delivery across the region.

## Data Availability

Data supporting this study are not publicly available but can be accessed upon request. Please contact alb@gu.se
